# Three-Dimensional Engine-Based Geometric Model Optimization Algorithm for BIM Visualization with Augmented Reality

**DOI:** 10.3390/s22197622

**Published:** 2022-10-08

**Authors:** Pa Pa Win Aung, Woonggyu Choi, Almo Senja Kulinan, Gichun Cha, Seunghee Park

**Affiliations:** 1 Department of Global Smart City, Sungkyunkwan University, Suwon 16419, Korea; 2 School of Civil, Architectural Engineering & Landscape Architecture, Sungkyunkwan University, Suwon 16419, Korea

**Keywords:** building information modeling (BIM), augmented reality (AR), mesh reconstruction, geometric model optimization, 3D engine

## Abstract

Building information modeling (BIM), a common technology contributing to information processing, is extensively applied in construction fields. BIM integration with augmented reality (AR) is flourishing in the construction industry, as it provides an effective solution for the lifecycle of a project. However, when applying BIM to AR data transfer, large and complicated models require large storage spaces, increase the model transfer time and data processing workload during rendering, and reduce visualization efficiency when using AR devices. The geometric optimization of the model using mesh reconstruction is a potential solution that can reduce the required storage while maintaining the shape of the components. In this study, a 3D engine-based mesh reconstruction algorithm that can pre-process BIM shape data and implement an AR-based full-size model is proposed, which is likely to increase the efficiency of decision making and project processing for construction management. As shown in the experimental validation, the proposed algorithm significantly reduces the number of vertices, triangles, and storage for geometric models while maintaining the overall shape. Moreover, the model elements and components of the optimized model have the same visual quality as the original model; thus, a high performance can be expected for BIM visualization in AR devices.

## 1. Introduction

Building information modeling (BIM) technology has been advanced to merge projects centered around a digital twin of information, which is needed for collaboration as projects become increasingly complicated [1]. BIM refers to building information management that can perform actions and processes to create objects and information, add and share information, and manage processes throughout the design, construction, and maintenance phases [2]. It is being used extensively in the construction and civil engineering fields, and attempts are being made to apply it at all stages before and after construction. BIM with geometric and semantic data allows users to virtually test alternative designs, anticipate problems that may arise during construction, and interact with each building component in a 3D space. BIM integration with augmented reality (AR) can also strengthen the visualization of different phases of a project’s lifecycle and ultimately increase the applicability of BIM in fieldwork [3]. Numerous methods have been proposed for integrating AR and BIM [4,5]. AR technology provides users with a real view that is augmented by combining digital and real information on a virtual screen, such as a tablet or HoloLens [6]. AR enables the real-time visualization of BIM information in real physical environments during the project, maintenance, and operational phases of buildings [7].

In the construction industry, a model built with BIM is used to efficiently manage collected data. Most models in the architecture, engineering, and construction (AEC) fields require large amounts of storage, owing to their irregular geometry, density of components, and wordiness of information. Construction site data are often digitized using BIM; however, owing to the considerable amount of data, it is often difficult to express the information in the model on a web-based platform or AR device environment. In addition, AR-based construction information visualization that connects the real and virtual worlds is essential to develop the construction industry through automation. To convert models to AR, they need to be converted to a neutral file format, such as IFC, FBX, or OBJ, for importing into the 3D engine. In BIM, the process of converting a large model to such a neutral file format needs the storage data size to be reduced to improve the data-loading speed, minimize dizziness in the visualization process, and implement the full size of the model. In addition, because of the size and complexity of the models, it takes a long time to transfer and a considerable computational effort to render. A mesh reconstruction algorithm or technique that can reduce geometric information is therefore needed.

**Figure 1:**
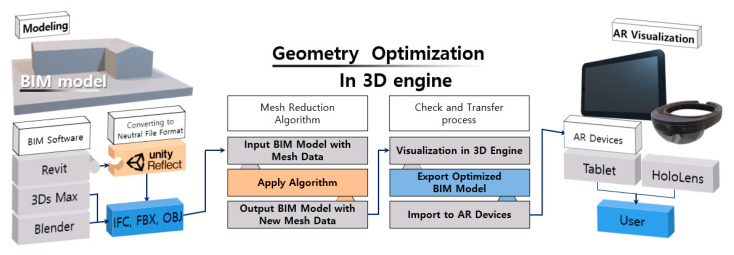
Conceptual framework of 3D engine-based geometry optimization for augmented reality (AR) visualization.

In this study, we used BIM and developed a 3D engine-based geometric optimization algorithm that can visualize models in AR. The conceptual framework of the proposed methodology is illustrated in Figure 1, and consists of three main procedures: (1) modeling, (2) geometry optimization in the 3D engine, and (3) AR visualization. In the modeling section, Revit software is used for converting the 2D drawings into 3D models and exporting them to an FBX file format for transmission into the 3D engine. After that, the BIM model containing mesh data is imported into the 3D engine, and the mesh reconstruction algorithm, which was developed based on the barycentric coordinates-based triangle centroid formula, is applied by reconstructing the mesh of the model for optimization. Then, the optimized model is visually checked within the 3D engine, built into the AR device, and visualized in the AR environment. The major contribution of this study is the implementation of a triangular mesh reconstruction approach in a 3D engine for augmented reality visualization. The algorithm is capable of pre-processing BIM shape data and of realizing an AR-based full-size model, which will improve the efficiency of decision making and project processing for construction management.

### BIM-AR Integration

BIM can be used to support better decision making at all stages of project management by facilitating information exchange and interoperability in digital form. In addition, it can be used to maintain data about building facilities for an overall project [8]. As a result, BIM has recently attracted increased attention in the AEC field. Furthermore, AR has been widely applied in the AEC field to better visualize 3D models created using BIM software for over ten years [9]. AR merges digital data with the real world, enabling a clearer visualization of projects, better planning and decision making, better component assembly, and an improved monitoring of facilities. AR in architecture and engineering includes design reviews [10], structural analysis [11], and daylight analysis [12]. At construction sites, AR can assist in site inspections [13], construction simulations [14], and safety management [15]. In other fields, such as operations and maintenance, AR has been implemented for emergency evacuation [16] and facility maintenance management [17].

The integration of BIM and AR has been investigated to improve the visualization of 3D models. It has been explored in several different fields in the construction industry [18,19,20]. The research areas include on-site construction process control [21], construction safety management and visualization [22], and construction collaboration and discussions for multiple users [23]. May et al. (2022) [24] developed an on-site BIM-based AR defect management system for construction inspections. Garbett et al. (2021) [25] proposed a multi-user collaborative BIM-AR system that has the ability to view, interact, and collaborate with 3D and 2D BIM data via AR. However, efficient model optimization methods that simplify geometric data are still needed to improve the capability of model delivery and operation.

## 2. Background

### 2.1. Model Optimization Methods

**Figure 2:**
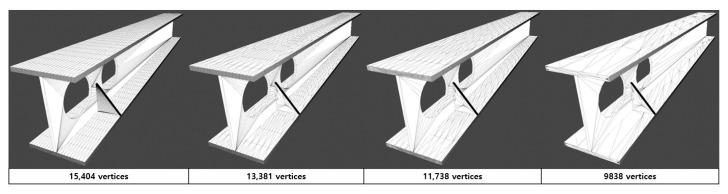
Sample of mesh optimization process.

Polygonal modeling is an approach for modeling objects by illustrating or approximating their surfaces using polygon meshes [26]. Polygon models are extensively used in several areas of computer graphics, such as movies, computer games, and advertisements. The complexity of these models with respect to the number of polygons poses a major challenge to the computing power of the hardware. Most models used in the AEC field are particularly large and complicated. It is quite normal for a single building model to have many components, and if multiple buildings are included, the number of polygons can be extremely large, as the number of components will inevitably be higher. Processing this type of model without optimization entails an enormous computational workload for the hardware. Model optimization methods can provide developers with solutions that simplify models by simplifying the number of polygons, which reduces the data processing load for the hardware. Polygonal optimization methods provide a solution for developers struggling with complex models, as shown in Figure 2. These technologies simplify the polygonal geometry of the insignificant parts of the model without significant loss to the visual content of the scene.

Many different mechanisms have been developed for polygon reduction; the four primary polygon removal mechanisms are vertex merging, sampling, adaptive subdivision, and decimation. Hoppe [27] introduced progressive meshes as the first dynamic optimization algorithm for general polygonal reduction by defining edge collapse, split, and swap mesh transformations. The surface simplification algorithm developed by Garland and Heckbert [28] uses quadric error metrics (QEM) to contract arbitrary vertex pairs that do not need to be connected by an edge. A vertex clustering method was carried out by placing a 3D grid on top of the model to reduce the number of polygons and reduce all the vertices within each cell to the most significant vertices within the cells [29]. There are other approaches for model optimization, such as sampling [30] and adaptive subdivision [31]; however, most existing model optimization methods are complicated and have a limited ability to properly address meshes while preserving the basic shape of the 3D model.

#### 2.1.1. Quadrics Error Metrics Method

Garland and Heckbert developed the QEM algorithm [28] and proposed a quadric error method. This algorithm focuses on the iterative contraction process of vertex pairs and assigns error quadrics. The error at a vertex is defined as $v={\left[v_{x},\;v_{y},\;v_{z},\;1\right]}^{T}$ for vertices in three dimensions and is the sum of the squared distances of v and its associated planes (v). To characterize the error at each vertex in order to define the cost of the contraction by choosing which contraction to perform during a given iteration, a symmetric 4 × 4 matrix Q is connected to each vertex, and the folding cost of the model is calculated in the quadratic form $\Delta \left(v\right)=v^{T}Qv$. The plane is represented as $p={\left[a\;b\;c\;d\right]}^{T}$ and defined by the equation in three-dimensional space ax+by+cz+d=0, where $a^{2}+b^{2}+c^{2}=1\;\mathrm{and}\;d$ is a constant. The vertex error can be defined as the sum of the squares of the distance to its plane [32]:

(1) $$\Delta \left(v\right)=\Delta (\left[v_{x}\;v_{y}\;v_{z}\;1{]}^{T}\right)=\underset{p\in planes\left(v\right)}{\sum }{\left(p^{T}v\right)}^{2}$$

where $K_{p\;}$, its related triangle set, is the fundamental error quadric of p:

(2) $$K_{p\;}=pp^{T}=\left|\begin{array}{cccc} a^{2} & ab & ac & ad\\ ab & b^{2} & bc & bd\\ ac & bc & c^{2} & cd\\ ad & bd & cd & d^{2} \end{array}\right|$$

The quadratic error matrix of the vertex v is $Q_{v}=\underset{p\in planes\left(v\right)}{\sum }K_{p}$. $Q_{1}+Q_{2}$ is the quadratic error matrix of the new vertex v’, where $\Delta \left(v^{\prime }\right)$ is the error cost of the new vertex v, and the folding cost in the edge-folding operation is $\Delta \left(v^{\prime }\right)=v^{T}\left(Q_{1}+Q_{2}\right)v$.

#### 2.1.2. Curvature Calculation-based Check Method for Border Mesh

It is important to consider the shape of the boundary curve to obtain the best boundary constraints. A better approximation of some vertices on the inner surface can be maintained by removing the vertices of the linear boundary. Although the QEM algorithm developed by Garland and Heckbert [28] is an approach for preserving important boundary edges by marking them during the initialization process, it is not without its own limitations. Their algorithm excessively simplifies the mesh with boundary constraints because it removes most of the surface edges and vertices before collapsing any boundary vertices. Bahirat et al. [33] advanced the curvature-based boundary preservation, which was inspired by the original QEM mesh simplification approach. They considered boundary vertex $v_{1}$ and its adjoining boundary vertices $v_{2}$ and $v_{3}$, because every boundary vertex has exactly two adjacent boundary vertices.

Using this approach [34], Goldman computed the curvature of the boundary curve *k* at vertex $\;v_{1}$. Adding the quadratic of the boundary constraint plane to both endpoints of the boundary edge achieved a better approximation across the surface edges by removing some vertices of the linear bounds with Equation (3), where *Q*(*v*_1_) and *Q*(*v*_2_) are the quadrics related to vertices *v*_1_ and *v*_2_, respectively; *W_b_* is a user-defined weight factor; *k*(*v*_1_) and *k*(*v*_2_) are the curvatures of the boundary at vertices *v*_1_ and *v*_2_, respectively; and *Q_bcp_* is a quadric of the boundary constraint plane at the edge (*v*_1_,*v*_2_).

(3) $$\begin{array}{c} Q\left(v_{1}\right)=Q\left(v_{1}\right)+W_{b}\;\times k\left(v_{1}\right)\times Q_{bcp}\\ Q\left(v_{2}\right)=Q\left(v_{2}\right)+W_{b}\times k\left(v_{2}\right)\times Q_{bcp} \end{array}$$

The quadratic error metric (QEM) approaches with the insight that the most appropriate boundary edges can be reduced while lying along the curvature were referenced for this study. Furthermore, the barycentric coordinate-based triangular centroid method was utilized in the 3D engine-based mesh reconstruction method, which is designed explicitly for AR visualization to reduce the number of vertices and reconstruct the triangle mesh of BIM for geometric optimization.

## 3. Materials and Methods

### 3.1. Geometric Model Optimization for BIM

Geometric optimization is an important step in the AEC model optimization process, which refers to deleting or modifying the meshes of a model and aims to reduce the size of the model while maintaining the original shape. A neutral file format, such as IFC, FBX, or OBJ, is required to import a model into the 3D engine. Most AEC models are created using BIM software and can be exported to a neutral file format successfully. Models created with BIM in 3D engines, such as Unreal Engine and Unity, are expressed with a surface modeling method. These methods represent a 3D solid using polygonal meshes consisting of triangles and vertices, unlike the solid BIM methods, such as boundary representation and constructive solid geometry. The file format of the imported model is not affected by the representation of models in 3D engines because, after being imported, the model is expressed by polygonal meshes, regardless of the format of the transport file. This study focused only on the mesh reconstruction of models created with BIM consisting of triangle-based polygonal meshes used in 3D engines.

#### 3.1.1. Mesh Reconstruction Method

**Figure 3:**
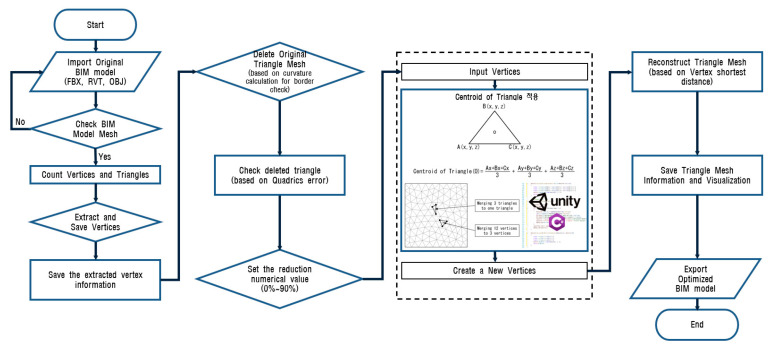
Flowchart of mesh reconstruction algorithm.

The mesh reconstruction algorithm was designed to generate a low-poly version of a 3D model effectively and accurately, after considering the different properties of the model used by the 3D engine. The proposed algorithm was extended using the primary QEM method developed by Garland and Heckbert [28,32]. The overall process of the mesh reconstruction method is illustrated in Figure 3. The proposed approach concentrates only on the optimization of the mesh geometry by extracting the vertices and reconstructing the triangles of the meshes. The algorithm operates according to the following steps.

**Figure 4:**
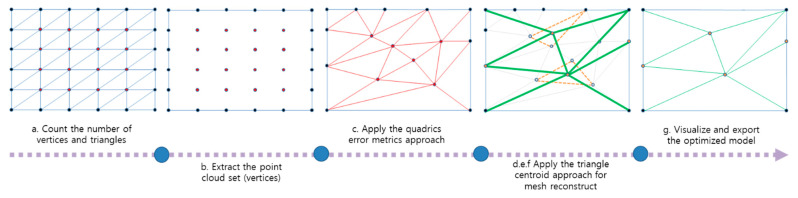
Illustration of algorithm steps.

(a) Examine the geometric mesh information of the imported original model using 3D engine properties and count the number of vertices and triangles of the model.

(b) Separate the shape information by extracting only the point cloud set (vertices) from the mesh configuration information constituting the surface, conforming to geometric and topological properties, and excluding non-shrinkable edges.

(c) Delete the original triangular meshes and check the deleted triangular meshes based on curvature calculation and the QEM method.

(d) Reduce the existing vertices so that the extracted point cloud set applies the barycentric coordinate-based triangle centroid formula in a 3D engine, such as the Unity code, with C# to create new vertices.

(e) Adjust the reduction in numerical value (up to 90%). Repeat this process based on the set reduction value.

(f) When the reduction value has been sufficiently satisfied, configure and reconstruct the triangular mesh with the newly created vertex information.

(g) Perform the visualization and exportation of the optimized model. An explanation of the sample shape of the algorithm steps is presented in Figure 4.

The proposed algorithm was developed as a reconstruction technique, using the barycentric coordinate-based triangle centroid formula via calculations that take the average of the x-, y-, and z-coordinates of the triangle vertices in order to merge at least three triangles into one triangle.

#### 3.1.2. Barycentric Coordinates-Based Triangle Centroid Formula

**Figure 5:**
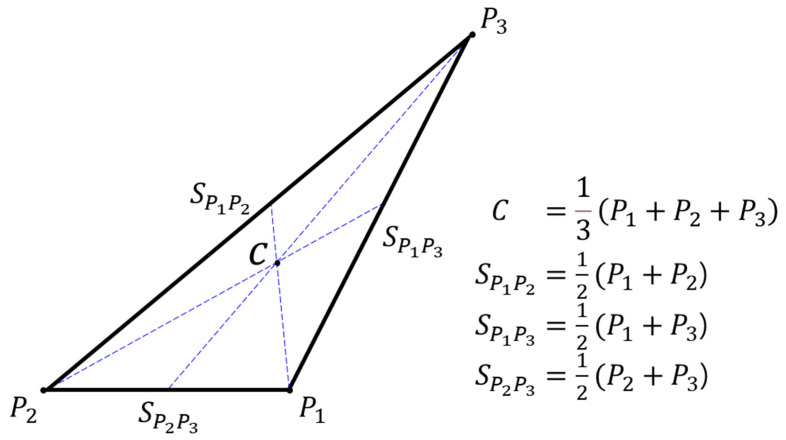
Triangle centroid formulation by Abraham Albert Ungar in August 2010 [35].

The center of the triangle C is at the intersection of the triangular midlines [35], as shown in Figure 5. Let $P_{1}P_{2}P_{3}$ be a triangle with vertices $P_{1},P_{2},\;\mathrm{and}\;P_{3}$ in Euclidean *n*-space $R^{n}$, and let C be the centroid of the triangle, as illustrated in Figure 5 for *n* = 2. Then, C is given in terms of its barycentric coordinates $\left(s_{1}:s_{2}:s_{3}\right)$ with respect to the set $\;\left\{P_{1},P_{2},P_{3}\right\}$ by the equation:

(4) $$C=\frac{s_{1}P_{1}+s_{2}P_{2}+s_{3}P_{3}}{s_{1}+s_{2}+s_{3}}$$

where the barycentric coordinates $\;m_{1}$, $m_{2}$, and $m_{3}$ of $P_{3}$ are determined in Equation (9) below. The midpoint of side $P_{1}P_{2}$ is given by:

(5) $$S_{P_{1}P_{2}}=\frac{P_{1}+P_{2}}{2}$$

Thus, an equation of the line $L_{123}$ through the points $S_{P_{1}P_{2}}$ and $P_{3}$ is

(6) $$L_{123}\left(t_{1}\right)=P_{3}+\left(-P_{3}+\frac{P_{1}+P_{2}}{2}\right)t_{1}$$

with the line parameter $t_{1}\;\in R.$

Line $L_{123}\left(t_{1}\right)$ contains one of the three medians of triangle $P_{1}P_{2}P_{3}$. Invoking cyclicity, equations of lines $L_{123},\;L_{231},$ and $L_{312}$, which contain the three triangle medians, respectively, are acquired from Equation (6) by index cyclic permutations:

(7) $$\begin{array}{c} L_{123}\left(t_{1}\right)=\frac{t_{1}}{2}P_{1}+\frac{t_{1}}{2}P_{2}+\left(1-t_{1}\right)P_{3}\\ L_{231}\left(t_{2}\right)=\frac{t_{2}}{2}P_{2}+\frac{t_{2}}{2}P_{3}+\left(1-t_{2}\right)P_{1}\\ L_{312}\left(t_{3}\right)=\frac{t_{3}}{2}P_{3}+\frac{t_{3}}{2}P_{1}+\left(1-t_{3}\right)P_{2} \end{array}$$

where $t_{1},t_{2},t_{3}\in R$.

The triangle centroid C in Figure 5 is the simultaneous point of the three lines in Equation (7) above. The concurrency point is determined by solving Equation (7), where $L_{123}\left(t_{1}\right)=L_{231}\left(t_{2}\right)=L_{312}\left(t_{3}\right)$ for unknown $t_{1},t_{2},t_{3}\in R,$ giving rise to $t_{1}=t_{2}=t_{3}=2/3$. Hence, C is given by the equation:

(8) $$C=\frac{P_{1}+P_{2}+P_{3}}{3}$$

Comparing Equations (8) and (4), we find that the special barycentric coordinates $\left(s_{1},s_{2},s_{3}\right)$ of C with respect to the set $\left\{P_{1},P_{2},P_{3}\right\}$ are given by $s_{1}=s_{2}=s_{3}=1/3$. Hence, the convenient barycentric coordinates $\left(s_{1}:s_{2}:s_{3}\right)$ of C are given by

(9) $$\left(s_{1}:s_{2}:s_{3}\right)=\left(1:1:1\right)$$

The same expression was reported by Kimberling [36], among others.

The proposed mesh reconstruction algorithm was recondited in a script format to facilitate the development process and applied to the Unity 3D engine [37,38]. Scripts are written in the C# language, which Unity can understand.

### 3.2. Test-Bed Specifications and 3D Modeling

Two types of model experiments were performed to demonstrate the efficiency, reliability, and quality of the proposed method.

**Figure 6:**
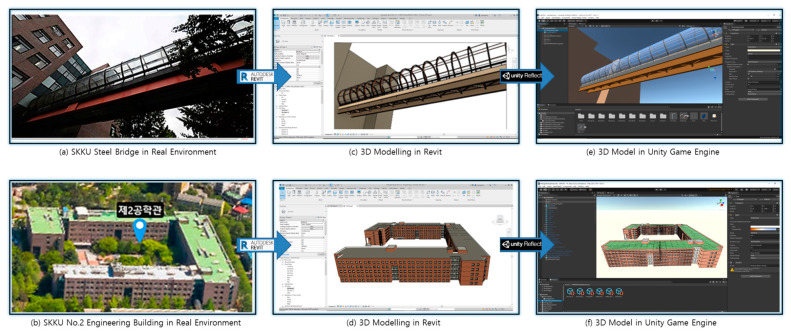
Three-dimensional modeling process for SKKU steel bridge and No. 2 engineering building. Real environment: (**a**) bridge, and (**b**) building. Model in Revit: (**c**) bridge, and (**d**) building. Model in Unity: (**e**) bridge, and (**f**) building.

The first model is a steel glass structure bridge (length = 34 m, height = 5 m, width = 2 m) located between No. 1 engineering building 23 and No. 2 engineering building 25. The second model is No. 2 engineering building. Both test models were located at Sungkyunkwan University and were selected to demonstrate the validity of the proposed method. They were modeled with Autodesk Revit using existing two-dimensional drawings and exported to the Unity 3D engine using the Unity Reflect process. Unity Reflect can be leveraged as a plug-in for Autodesk Revit (BIM software) and can transfer models from Revit into a real-time 3D experience [39]. The process of the modeling for the two test models is shown in Figure 6.

## 4. Results

### 4.1. Validation of Mesh Reconstruction Method for Bridge

**Figure 7:**
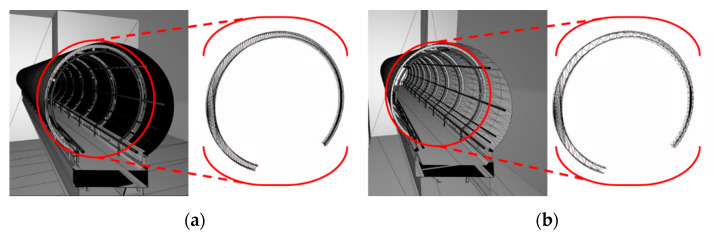
SKKU steel bridge models and internal glass structure (mesh-form): (**a**) original and (**b**) optimized.

The proposed mesh reconstruction algorithm was tested with the bridge model to validate the algorithm, particularly for the curve components. As shown in Figure 7, the entire bridge, including the different structures, was optimized using the proposed method, and it was confirmed that the shape of the entire model did not change. For comparison, three parameters were considered: they were the numbers of vertices and triangles in the relevant meshes and the file size of the models, which could indicate the intuitive reduction efficiency. Table 1 lists the results of the proposed mesh reconstruction method for the test bridge model. The numbers of vertices and triangles were reduced by 56.7% and 67.9%, respectively, and the file size was reduced by 73.4%.

### 4.2. Validation of Mesh Reconstruction Method for Building

**Figure 8:**
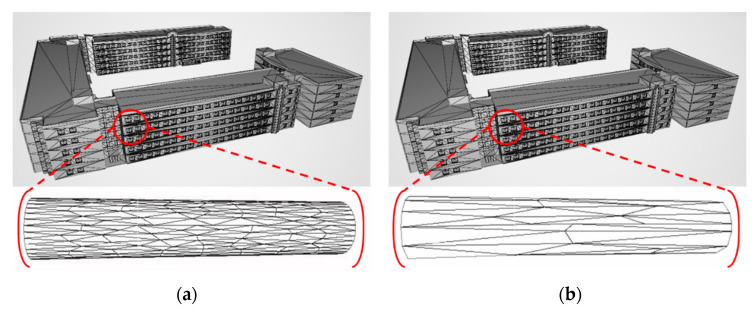
SKKU No. 2 engineering building models and internal round-column structure (mesh form): (**a**) original, and (**b**) optimized.

A building model was tested to verify the performance of the proposed algorithm. Figure 8 shows that the proposed algorithm can successfully optimize the entire building and its related facilities, and the overall appearance of the building components does not change significantly after optimization. The numbers of vertices, triangles, and the file sizes were compared for the model of the building and are shown in Table 2. The numbers of vertices and triangles were reduced by 46.6% and 58.6%, respectively, and the file size was reduced by 66%.

### 4.3. AR Visualization Test of the Optimized Models

**Figure 9:**
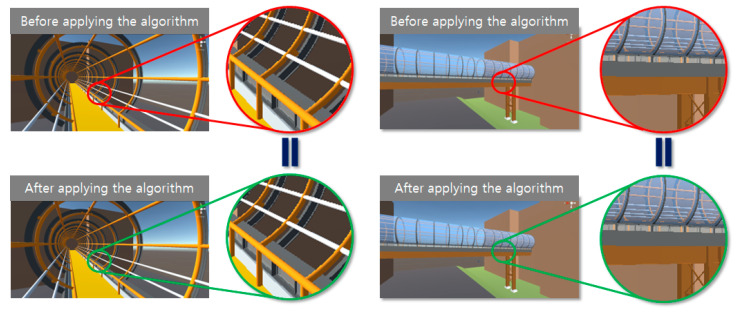
SKKU steel bridge in 3D engine before and after applying algorithm.

**Figure 10:**
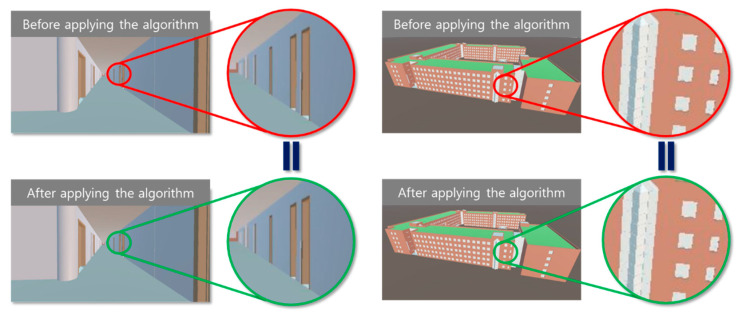
No. 2 SKKU engineering building in 3D engine before and after applying algorithm.

**Figure 11:**
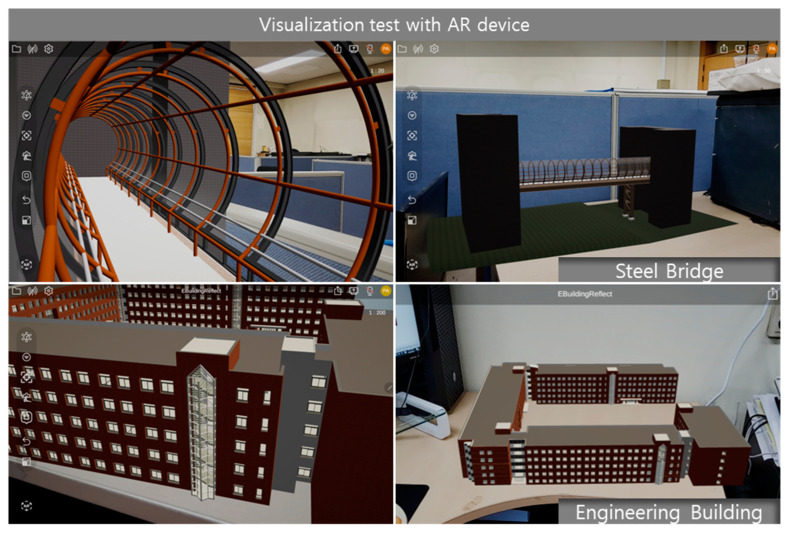
Optimized model visualization with AR device.

After the models were optimized using the proposed methods, both the original and optimized models were tested for visualization on the AR platform. The results show that there are no significant visual differences between the overall models as well as the internal components. This was confirmed in the 3D engine, as shown in Figure 9 and Figure 10. The results were successfully imported into the AR device, as shown in Figure 11.

Although BIM and AR technologies are being introduced in the AEC industry, there is a lack of apparatuses that can efficiently transmit data from BIM to AR. The complicated 3D models can reduce visualization efficiency, increase the computation task while contributing, and need more storage space when using AR devices. Therefore, a 3D engine-based mesh reconstruction method for high-performance visualization when using AR devices is developed in this paper. This new approach improves the shape preservation of the BIM model and thus reconstructs the model in as high a quality as possible. This study aimed to effectively visualize the BIM model with metadata by optimizing the geometric information of the model using the mesh reconstruction algorithm, and to improve the decision making and project processing efficiency of construction managers. Two different types of models were used to evaluate the overall performance of the developed method. The proposed method can significantly optimize BIM geometric models, reduce the file sizes of the models, and visualize BIM models with a high quality on AR devices.

## 5. Conclusions

This study addressed challenges experienced with data storage, data transfer, computer work/processing load, and geometric data display when applying BIM to large-capacity AEC projects. The basis of the solution for model storage optimization and display was constructed by managing new information storage and architecture. The architecture includes 3D engine-based geometric optimization with a mesh reconstruction method that extends the original QEM approach by utilizing a barycentric coordinate-based triangle centroid formula. The testing of the proposed method with a large steel bridge model and a building model was successfully accomplished, and it appeared that the volume of storage data could be reduced by 73.4% and 66%, respectively, without any significant effect on the quality of the visual end products. The key contribution to this level of achievement was the improvement introduced to the prior QEM-based mesh optimization method, with the introduction of surface division, accumulated error measurement, and the reconstruction of the triangle mesh. These improvements significantly reduce the number of triangles in the mesh while preserving the topology. The proposed method was successfully tested, and the number of meshes was greatly reduced, displaying a model of a very good quality which can be expected to increase the efficiency of decision making and project processing for construction managers.

For future work, more varied and complicated AEC model tests will be conducted to achieve better accuracy in mesh reconstruction. Moreover, experiments will be conducted on not only the optimized models, but also the semantic data visualization on AR devices for construction site management.

## Figures and Tables

**Table 1:** SKKU steel bridge original and optimized parameters.

Model	Category	Original	Optimized	Improvement (%)
SKKUsteel bridge	Number of vertices	590,942	255,625	↓56.7
	Number of triangles	335,564	107,573	↓67.9
	File size (KB)	50,535	13,451	↓73.4

**Table 2:** SKKU No. 2 engineering building original and optimized parameters.

Model	Category	Original	Optimized	Improvement (%)
SKKU No. 2 engineering building	Number of vertices	4,183,198	2,235,879	↓46.6
	Number of triangles	3,231,090	1,336,165	↓58.6
	File size (KB)	364,761	124,173	↓66.0

## Data Availability

This study did not report any data.
